# Longitudinal trends in screening males and females for intimate partner violence as part of a systemic multi-specialty health system intervention

**DOI:** 10.1186/s13104-021-05754-x

**Published:** 2021-09-03

**Authors:** Cari Jo Clark, Lynette M. Renner, Qi Wang, Nyla I. Flowers, Grace Morrow, Mary Logeais

**Affiliations:** 1 Rollins School of Public Health, Emory University, Atlanta, GA USA; 2 School of Social Work, University of Minnesota, St. Paul, MN USA; 3 Clinical and Translational Science Institute, University of Minnesota, Minneapolis, MN USA; 4 School of Medicine, University of Minnesota, Minneapolis, MN USA

**Keywords:** Intimate partner violence, Screening, Men, Victimization

## Abstract

**Objective:**

To assess intimate partner violence screening for males and females in a health system that underwent a systemic intervention to improve survivor identification and response. Electronic health record data from 13 clinics were accessed for February of 2017, 2018, and 2019 to calculate screening rates and positive screening rates for intimate partner violence by clinic and sex-race groups (n  =  11,693 non-Hispanic White females; n  =  4318 Other females; n  =  9184 non-Hispanic White males; n  =  3441 Other males). Linear mixed effects models were used to examine whether screening rates differed significantly over time and by sex-race group.

**Results:**

Screening rates were 31% for the first 2 years and 16% for 2019. Screening rates varied greatly by clinic. Dermatology, psychiatry, and otolaryngology clinics had average or above screening rates all 3 years. Differences in screening rates across sex-race groups were minimal. Average positive screen rates were 1.3%, 0.4%, and 2.6% in 2017, 2018, and 2019, respectively, with psychiatry having the highest positive screen rate. Positive screen rates were highest for non-Hispanic White females (3.5%). Universal screening in this health system was not yielding survivors comparable to existing estimates among clinic-based populations. Other identification approaches require testing to effectively identify survivors within the health sector.

## Introduction

Approximately one-third of adult females (36.4%) and males (33.6%) and in the United States have experienced physical violence, sexual violence, and/or stalking perpetrated by a romantic or sexual partner in their lifetime, with sexual and gender minorities having higher rates than average [1]. Research on intimate partner violence (IPV), mostly among females, has identified numerous adverse mental, behavioral, and physical health outcomes [2] and higher healthcare utilization even after the abuse has ended [3]. Given the potential negative outcomes of this highly prevalent social determinant of health, the health sector has been identified as an important gateway to secondary prevention and the uptake of supportive services.

Screening and ongoing support services for IPV among females of childbearing age is recommended by the US Preventive Services Task Force [4] but is infrequently provided in some settings. Screening and support services data for males were too scant for the Task Force to make a recommendation. However, a recently reported nationally representative survey of males aged 18–35 found that almost all respondents supported clinical inquiry about IPV victimization (92%) and perpetration (90%); and although 27% reported IPV victimization and 19% reported perpetration, only 13% of respondents reported having been asked about victimization and 11% about perpetration [5].

To address this gap, we examined the longitudinal change in IPV screening practices for adult females and males in a large, multi-specialty healthcare system in the Midwest that underwent a systemic intervention involving required online provider training; in-person follow-up training; a validated electronic health record screening tool; intranet-based referral information; and the provision of trained support within the health system and community-based agencies with the aim of improving the detection and response to IPV survivors [6].

## Main text

### Methods

#### Sample

The Clinics and Surgery Center is an ambulatory surgery center and multi-specialty medical facility that includes 37 clinics. Thirteen clinics serving the highest volumes of female patients were identified as the focus of this study including: blood and marrow transplant, cardiology, dermatology, otolaryngology, endocrinology, family practice, internal medicine, neurology, oncology, orthopedics, sports medicine, rheumatology, and psychiatry.

#### Intervention

A four-item screen was embedded in the electronic health record (EPIC™) using a flow sheet, which was visible to providers but did not show up in the after-visit summary, to assess the presence of IPV among adult patients. Its use was guided by a screening and response protocol and supported by required and optional trainings. Specifically, rooming staff and social workers received mandatory online and optional in-person training on the protocol. The online and in-person trainings were also available to all other providers, including physicians, nurses, and behavioral health clinicians. According to the protocol, at least once every three months (to provide multiple opportunities to disclose but not overburden patients being seen very frequently for treatment), all adult patients were to be screened by the rooming staff using the validated screening tool to assess past year victimization by a current partner or ex-partner. Patients were not screened if they were not alone, did not speak English and did not have a medical interpreter, if it had not been 3 months, or if they were under the age of 18. In-screen text guided the protocolized positive screen response process.

#### Data and analysis

Data on adult patients (18 years or older) were extracted from the electronic health record. Data elements included the date of service, patient sex, and the HARK [7] screening tool responses, which included four items assessing past year experiences of being: (1) humiliated or emotionally abused in other ways; (2) afraid; (3) forced to have any kind of sexual activity (original text used the word rape and this was changed with permission); and (4) kicked, hit, slapped or otherwise physically hurt by a current or former partner. An affirmative response to any of the items defined a positive IPV screen. The HARK is one of the few brief tools that capture three areas of IPV (sexual, physical, psychological) and also has strong psychometric properties [8], with sensitivity of 81% and specificity of 95% [7].

Screening rates were calculated by dividing the number of patients who were screened by the number of patients who had any visit to the study clinics over a two-year period represented by data from February 2017 (when all clinics had access to the screen), February 2018 and 2019, considering that patients were to be screened no more frequently than every 3 months (n  =  11,693 non-Hispanic White females; n  =  4318 Other females; n  =  9184 non-Hispanic White males; n  =  3441 Other males). Total IPV screening rates and positive screen rates (percent positive among those screened) were calculated for each clinic, separately by sex-race (non-Hispanic White vs. Other) groups, for each time period. Linear mixed effects models were used to examine whether screening rates differed significantly over time and by sex-race group, and pairwise comparisons adjusted for multiple comparisons were used to assess differences.

## Results

Overall, IPV screening rates were approximately 31% for the first 2 years and 16% for 2019 (Table 1). Notably, screening rates varied greatly by clinic (Table 2), but the overall trend, exemplified by 10 out of the 13 clinics, was a 2019 rate that was lower than both 2017 and 2018 rates. Dermatology, psychiatry, and otolaryngology clinics had average or above screening rates all 3 years.

**Table 1:** Screening rates by year and race (N = 28,636)

Year	Overall	Non-Hispanic White females (n = 11,693)		Other females (n = 4318)		Non-Hispanic White males (n = 9184)		Other males (n = 3441)		P value for difference by race
		Mean	SE	Mean	SE	Mean	SE	LS Mean	SE	
2017	31.0	32.9	5.2	31.4	5.2	31.9	5.3	27.9	4.6	0.03
2018	31.4	32.8	6.1	29.9	5.8	31.1	6.2	31.8	6.9	0.10
2019	16.4	17.2	4.6	18.0	4.7	15.4	4.6	14.8	4.8	0.45

Linear mixed models

*SE* standard error

**Table 2:** Percent of patients screened by race-sex groups, year and clinic (*N*  =  28,636)

Clinic	Non-Hispanic White females (n = 11,693)			Other females (n = 4318)			Non-Hispanic White males (n = 9184)			Other males (n = 3441)			P value for difference by race by year		
	2017	2018	2019	2017	2018	2019	2017	2018	2019	2017	2018	2019	2017	2018	2019
Blood and marrow transplant	4.5	14.6	2.4	12.9	5.1	7.7	3.8	14.3	3.8	10.2	14.9	5.8	0.09	0.45	0.33
Cardiology	38.4	3.7	7.1	31.0	4.5	5.7	37.7	5.6	0.4	35.0	5.2	0.0	0.69	0.75	< 0.01
Dermatology	69.0	63.7	26.2	76.9	56.0	32.8	66.8	62.8	17.1	63.6	58.2	26.1	0.17	0.42	< 0.01
Otolaryngology	32.3	66.3	20.7	27.4	56.3	25.3	33.3	69.3	19.8	31.3	77.5	17.5	0.75	0.02	0.59
Endocrinology	4.7	0.0	0.3	4.9	0.0	0.0	3.0	0.5	0.0	0.0	0.0	0.0	0.20	0.57	1.00
Family practice	45.6	24.6	47.3	40.0	15.8	42.9	47.4	32.0	53.3	37.0	10.3	58.3	0.82	0.11	0.63
Internal medicine	46.3	27.2	45.1	48.0	30.3	48.4	42.9	24.2	43.3	37.1	24.9	40.2	0.18	0.55	0.59
Neurology	27.9	57.7	0.0	34.4	61.1	0.0	23.8	53.3	0.0	16.1	54.0	0.0	0.10	0.57	1.00
Oncology	24.5	33.2	35.5	23.1	35.0	37.6	26.6	31.6	27.1	25.6	51.2	16.7	0.85	0.01	< 0.01
Orthopedics	7.5	11.7	0.2	6.7	9.8	0.0	7.9	6.9	0.0	6.1	6.5	0.0	0.90	0.06	0.67
Sports medicine	27.2	22.4	8.1	18.5	22.4	8.1	22.8	14.2	8.8	21.4	11.5	6.4	0.49	0.03	0.92
Rheumatology	50.3	51.8	6.2	46.7	46.9	2.3	44.6	31.3	6.0	41.2	55.6	0.0	0.80	0.10	0.62
Psychiatry	49.0	50.0	24.9	37.9	45.0	23.7	54.5	58.3	20.4	37.5	43.8	21.2	< 0.01	0.01	0.52
Overall	32.9	32.8	17.2	31.4	29.9	18.0	31.9	31.1	15.4	27.9	31.8	14.8	0.03	0.10	0.45
P-value for differences across clinics with year	< 0.01	< 0.01	< 0.01	< 0.01	< 0.01	< 0.01	< 0.01	< 0.01	< 0.01	< 0.01	< 0.01	< 0.01			

**Fig. 1:**
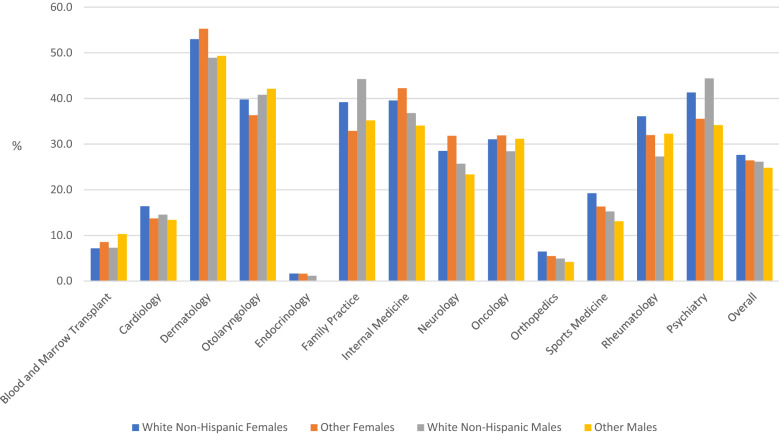
Average screening rates by race-sex group and clinic (N = 28,636)

Overall differences in screening rates across sex-race groups were detectable only in 2017 as males who identified as Other race had a slightly, but significantly lower screening rates (Table 1). Although some differences in screening rates were detectable in select clinics and years (Table 2), these periodic differences are overshadowed by a general consistency in screening practices across sex-race groups within clinics when averaged across years (Fig. 1), especially when compared to screening rate differences across clinics.

Across all clinics, the average positive screen rates were 1.3%, 0.4%, and 2.6% in 2017, 2018, and 2019, respectively, with psychiatry having the highest positive screen rate at 4.3%, 4.2%, and 6.7%, respectively. Positive screen rates were higher on average for non-Hispanic White females (3.5%) and Other females (1.4%) compared to non-Hispanic White males (0.3%) and Other males (0.5%).

## Discussion

The intervention demonstrated that an intensive systemic intervention can induce screening rates 70% and higher, including within clinics that are not traditionally the site of screening interventions. Shared resources and operational infrastructure streamlined implementation and facilitated a coordinated effort across a large system. Although the screening rates did not differ considerably across sex and race groups, screening rates declined significantly over time in most clinics and varied considerably by clinic. The decline in screening rates, particularly in the third year, was largely impacted by significant staff turnover in the health system and within the community-based service provider, coupled with a less intensive focus on retraining efforts. There was also variability in institutional leadership structure and support during the intervention period across clinics, which was detectable through the process evaluation. Collectively, these issues hindered a consistent response and level of engagement by staff, [9] therein highlighting the challenges to sustaining a systemic intervention, particularly when the full extent of the intervention is not mandated for accreditation nor sustainably funded through typical reimbursement schemes.

Based on an implementation analysis, health providers in this study reported benefit from the screening and response protocol on individual patient care and well-being [9]. A prior analysis of health record data also found that that utilization of behavioral health and social work services among screened survivors of IPV who accepted support was higher than among a comparable sample of screened survivors who did not accept support, adjusting for health status and violence severity [10]; however, other forms of healthcare utilization did not concomitantly decrease over the 2-year time frame of that analysis. Although the intervention resulted in sustained, annual training for the health system providers and the identification and support services around victimization were greatly enhanced, a more targeted, cost-effective approach is likely needed to identify survivors combined with a longer timeframe to assess potential health system benefits of intervening on this complex social determinant of health.

It often takes several inquiries for a patient to build trust toward a healthcare provider and disclose violence, but talking with a health care provider about the abuse has been found to increase women’s likelihood of using an intervention [11]. In our study, the low positive screen rate over the three-year intervention, especially for males and in most clinics included in this study, suggests that universal screening in this health system was not yielding survivors relative to estimates of their prevalence in clinic-based populations. Other methods of identification, such as case finding that is recommended by the World Health Organization, or universal education which shifts the emphasis from disclosure to education might better identify survivors [12, 13].

Although female survivors more frequently report harm from partner violence, 1 in 10 male survivors in the United States report harm from IPV victimization [1], and recent evidence suggests that males are not asked about their victimization and even fewer are asked about perpetration. The same nationally representative survey found that 90% of surveyed males supported clinical inquiry about perpetration, suggesting a potential willingness to engage in these conversations in the health sector. In our study, males were screened almost as frequently as females suggesting that it can be done at scale. The challenge is identifying the best route to identifying survivors of all sexes, gender identities, races and ethnicities to open the gateway to supportive services and fully realize the benefits of a health sector intervention.

## Limitations

Although racial differences could be examined, the patient population was predominantly non-Hispanic White, which is consistent with the population demographics of the state. Further, the small number of patients identifying neither as male or female precluded their inclusion in this analysis. Due to the large study setting size and heterogeneity among clinic sites, variations in staffing and rooming norms (i.e., getting patients alone), clinic processes and protocol adherence may have negatively influenced the number of patients who were screened, along with the lack of pop-up notification of screen eligibility based on the three-month interval. There was significant staff and leadership turnover throughout the intervention period. Competing patient care priorities and variation in scope of practice across the clinics likely contributed to under screening. Notably, this screening tool was one of many assessments required for nursing staff to complete, and likely the most time consuming, requiring sensitive conversation and more extensive documentation than other assessments, in addition to outreach for positive screens. In a prior analysis of study data on referrals made to individuals with a positive screen [10], 39.48% had missing data in the health record for the method of follow-up, providing evidence of under-documentation. Although the extent of under-documentation is unknown for the IPV screen, its impact would be to underestimate the screening rates detected in this study.

## Data Availability

Access to the clinical data repository and informatics support is restricted to researchers at the University of Minnesota, Fairview, and University of Minnesota Physicians, and is provided through the Best Practice Integrated Informatics Core (BPIC). The algorithm that was used to generate the dataset and the statistical programs used to compute the findings are available upon reasonable request from the corresponding author.

## Abbreviation

IPV: Intimate partner violence
